# Preparation of micro/nanopatterned gelatins crosslinked with genipin for biocompatible dental implants

**DOI:** 10.3762/bjnano.9.165

**Published:** 2018-06-11

**Authors:** Reika Makita, Tsukasa Akasaka, Seiichi Tamagawa, Yasuhiro Yoshida, Saori Miyata, Hirofumi Miyaji, Tsutomu Sugaya

**Affiliations:** 1 Department of Periodontology and Endodontology, Graduate School of Dental Medicine, Hokkaido University, Sapporo 060-8586, Japan; 2 Department of Biomaterials and Bioengineering, Faculty of Dental Medicine, Hokkaido University, Sapporo 060-8586, Japan; 3 School of Dental Medicine, Hokkaido University, Sapporo 060-8586, Japan; 4 Department of Periodontology and Endodontology, Faculty of Dental Medicine, Hokkaido University, Sapporo 060-8586, Japan

**Keywords:** cell attachment, cell proliferation, dental implants, gelatin, genipin, nanopatterning

## Abstract

**Background:** Collagen is a basic component of the periodontium and plays an important role in the function of the periodontal unit. Therefore, coating with collagen/gelatin has been applied to enable dental implants to positively interact with peri-implant tissues. Although the micro/nanoscale topography is an important property of the surface of dental implants, smaller collagen/gelatin surface patterns have not been sufficiently developed. Furthermore, only few reports on the behavior of cells on gelatin surfaces with different patterns and sizes exist. In this study, we developed micro/nanometer-scaled gelatin surfaces using genipin crosslinking, with the aim of understanding the use of patterning in surface modification of dental implants.

**Results:** Grooves, holes, and pillars, with widths or diameters of 2 µm, 1 µm, or 500 nm were fabricated using a combination of molding and genipin crosslinking of gelatin. The stability of the different gelatin patterns could be controlled by the degree of genipin crosslinking. The gelatin patterns at 20 mM concentration of genipin and 41% crosslinking maintained a stable, patterned shape for at least 14 days in a cell culture medium. A cell morphology study showed that the cells on groves were aligned along the direction of the grooves. In contrast, the cells on pillars and holes exhibited randomly elongated filopodia. The vinculin spots of the cells were observed on the top of ridges and pillars or the upper surface of holes. The results of a cell attachment assay showed that the number of surface-attached cells increased with increasing patterning of the gelatin surface. Unlike the cell attachment assay, the results of a cell proliferation assay showed that Saos-2 cells prefer grooves with diameters of approximately 2 µm and 1 µm and pillars with diameters of 1 µm and heights of 500 nm. The number of cells on pillars with heights of 2 µm was larger than those of the other gelatin surface patterns tested.

**Conclusion:** These data support that a detailed design of the gelatin surface pattern can control both cell attachment and proliferation of Saos-2 cells. Thus, gelatin surfaces patterned using genipin crosslinking are now an available option for biocompatible material patterning.

## Introduction

Topography on the micro- and nanoscale is an important property of the surface of biomaterials. Surface topographical patterns significantly affect cell adhesion, spreading, morphology, proliferation, and differentiation [1–5]. Surfaces with specific micro/nanopatterns have been developed in order to reduce platelet response [6], to regulate stem cell differentiation [7], to functionalize implant surfaces [8–9], and to prevent the formation of bacterial biofilms [10]. In the dental field, we have used different micro/nanopatterns that employ an apatite paste [11], a flowable composite resin [12], a titanium coat [13], and curable dental materials [14]. The behavior of cells on surfaces with different patterns can be altered by designing different surface patterns, and by the selection of different types of dental- and bio-materials. In the dental field, there is a huge need to develop surfaces with different patterns, either using biomaterials composed of natural tooth and periodontium, or their biomimetic equivalents.

Collagen is a basic component of the periodontium and plays an important role in the function of the periodontal unit [15]. Bundles of collagen fibers in the periodontal ligament, including Sharpey’s fibers, are vertically arranged from the surface of the tooth to the alveolar bone by their position and orientation. The resulting periodontal ligament fibers exhibit micro/nanopatterns arising as a result of the shape of bundles of collagen fibers [16–18]. Thus, coating surfaces with collagen has been used for dental implants to allow them to positively interact with peri-implant tissues [19–20]. Gelatin is derived from collagen through the process of controlled hydrolysis and is less immunogenic and economically more convenient than collagen. Because gelatin has a high cellular affinity, it is applied for the coating of dental implants [21–22]. It is also used as an absorbable hemostatic sponge to provide an occlusive matrix [23–24] and as a bone healing material in tissue engineering [25–26] in the field of dentistry. Recent studies have attempted to regenerate collagen fibers, lost as a result of periodontal disease, using topographical scaffolds [27–29].

Different patterns of coated collagen/gelatin have been used to control cell attachment and subsequent cellular function. Numerous methods have been used to control the patterning of collagen/gelatin surfaces. These include, polymerization of gelatin methacrylate (GelMA) [30], coating on patterned substrates [31–33], self-assembly of collagen fibers [34], and crosslinking, using glutaraldehyde [35], formaldehyde [36], carbodiimide [37], genipin [38–39], riboflavin [40], transglutaminase [41], silane coupling agents [42], as well as thermal dehydration [43–44]. Although the collagen/gelatin surface patterns that can be formed are in the micro- to nanoscale range, it is more difficult to fabricate surface patterns with smaller elements because of the solubility, swelling properties, and weakness of collagen/gelatin compared to engineered polymers. The type of surface patterning material or crosslinking agent used can also subsequently influence cytotoxicity and cell behavior [32–33].

Rizwan et al. have reported that micro/nanopillars comprised of gelatin could be fabricated through a combination of molding and polymerization of the gelatin with methacrylate [45]. These micro/nanopillars, with diameters of 1 µm or 250 nm, increased the proliferation of endothelial cells better than a planar gelatin surface. Zorlutuna et al. have reported that a collagen surface with a nanogroove could be fabricated by crosslinking with carbodiimide [37,46]. This nanogroove, with widths ranging from 333 to 650 nm, increased cell proliferation, as well as the initial cell attachment of smooth muscle cells, better than a planar collagen surface. These findings indicate that cell type, as well as pattern shape and size, can influence the efficiency of cell attachment and proliferation. Although the interactions between cells and patterned surfaces are complex, it is clear that the design of a material surface with optimal topological and chemical features can control cell behavior.

Genipin has been used as a naturally occurring crosslinking agent for the fixation of biological tissues and for the reinforcement of scaffolds. Genipin has low toxicity, being about 10,000 times less cytotoxic than other crosslinking agents, such as glutaraldehyde [47]. Crosslinking with genipin has been shown to result in an improvement in tensile strength, elastic modulus, and the solubility of collagen/gelatin scaffolds. Gelatin crosslinked with low concentrations of genipin has been shown to be biodegradable [48–49]. Islam et al. have previously reported that highly porous patterned collagen scaffolds, with porosity of 0.8 to 1.5 mm, could be fabricated by crosslinking with genipin [39]. Nadeem et al. have also reported that three-dimensional calcium phosphate/gelatin composite scaffolds, with an integrated surface pattern, could be fabricated by crosslinking with genipin [38]. These calcium phosphate/gelatin composite scaffolds could be fabricated with 40 µm pits, or 50 µm grooves, with lattice scaffolds that were multilayered as a result of the crosslinking. To date, collagen/gelatin surface patterns created by genipin crosslinking have been fabricated with pattern sizes ranging from 40 µm to 1.5 mm. However, smaller collagen/gelatin surface patterns, with sizes in the micro- to nanoscale range, have not yet been fabricated using crosslinking with genipin. Furthermore, there have only been a few reports comparing cell attachment and proliferation onto gelatin surfaces with different patterns and sizes, especially micro- or nano-sized grooves, holes, and pillars.

In this study, we have successfully fabricated gelatin patterns using crosslinking with genipin on both the micro- and nanoscales. Grooves, holes, and pillars, with widths or diameters of 2 µm, 1 µm, or 500 nm were fabricated using a combination of molding and genipin crosslinking of the gelatin. Following this, cell attachment and proliferation on the resulting gelatin surface patterns were assessed using human osteoblastic Saos-2 cells, with the aim of understanding the use of these gelatin patterned surfaces in surface modification of dental implants. Cell attachment increased as a result of gelatin patterning, compared to a planar surface. Furthermore, the degree of cell proliferation was very dependent on the shape and size of the different gelatin patterns.

## Results

### Preparation of gelatin patterns

**Figure 1:**
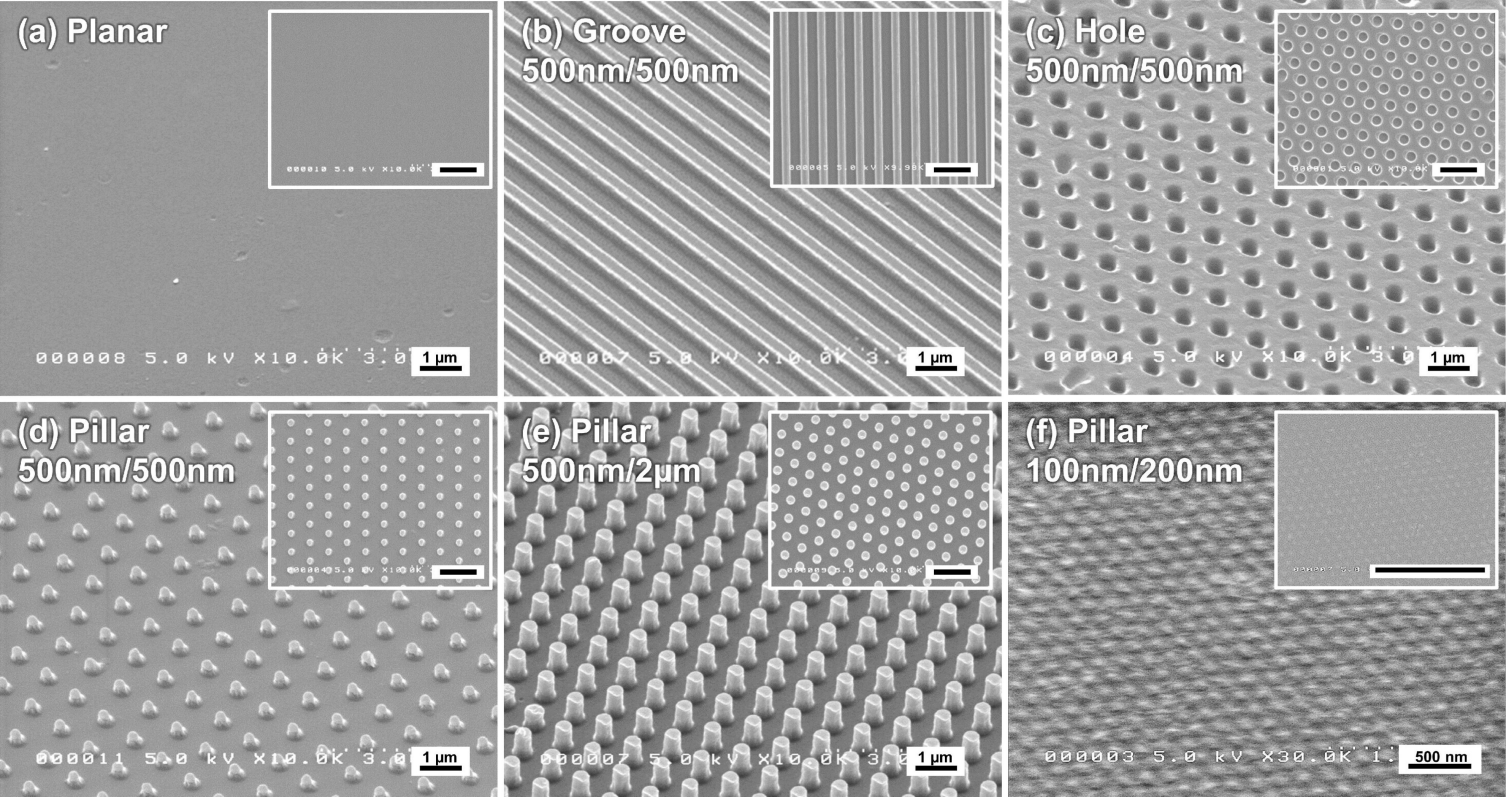
SEM images of gelatin crosslinked with 20 mM genipin during the molding of different surface patterns. The surface patterns were (a) a planar surface, (b) grooves, (c) holes, and (d) pillars molded to have a diameter of 500 nm and a height of 500 nm. (e) Pillars molded to have a diameter of 500 nm diameter and a height of 2 µm. (f) Pillars molded to have a diameter of 100 nm diameter and a height of 200 nm. The black scale bar in the insets represents 2 µm.

We first prepared different gelatin surface patterns using crosslinking with 20 mM genipin. The patterned surfaces were observed using a scanning electron microscope (SEM), as shown in Figure 1. In general, the grooves, holes, and pillars, designed to be 500 nm in diameter and 500 nm in height, were roughly transferred from the corresponding mold (Figure 1b to Figure 1d). The actual diameter of the features was determined from the SEM images. The width of the concave grooves and diameter of the holes were approximately 600 nm, indicating an enlargement of 1.2 times from the 500 nm diameter mold. The width of the convex ridges and the diameter of pillars were approximately 400 nm, indicating a shrinkage of 0.8 times from the 500 nm diameter mold. These data indicate that the volume of the gelatin patterns decreased slightly during the drying process, presumably as a result of the high water content of the 20 wt % gelatin solution. Although pillar patterns with a diameter of 500 nm, and either a height of 500 nm or 2 µm, were roughly transferred (Figure 1d and Figure 1e), pillar patterns molded with a 100 nm diameter and a 200 nm height were not successfully transferred (Figure 1f).

### The effect of genipin concentration on the stability of gelatin patterns in culture medium

**Figure 2:**
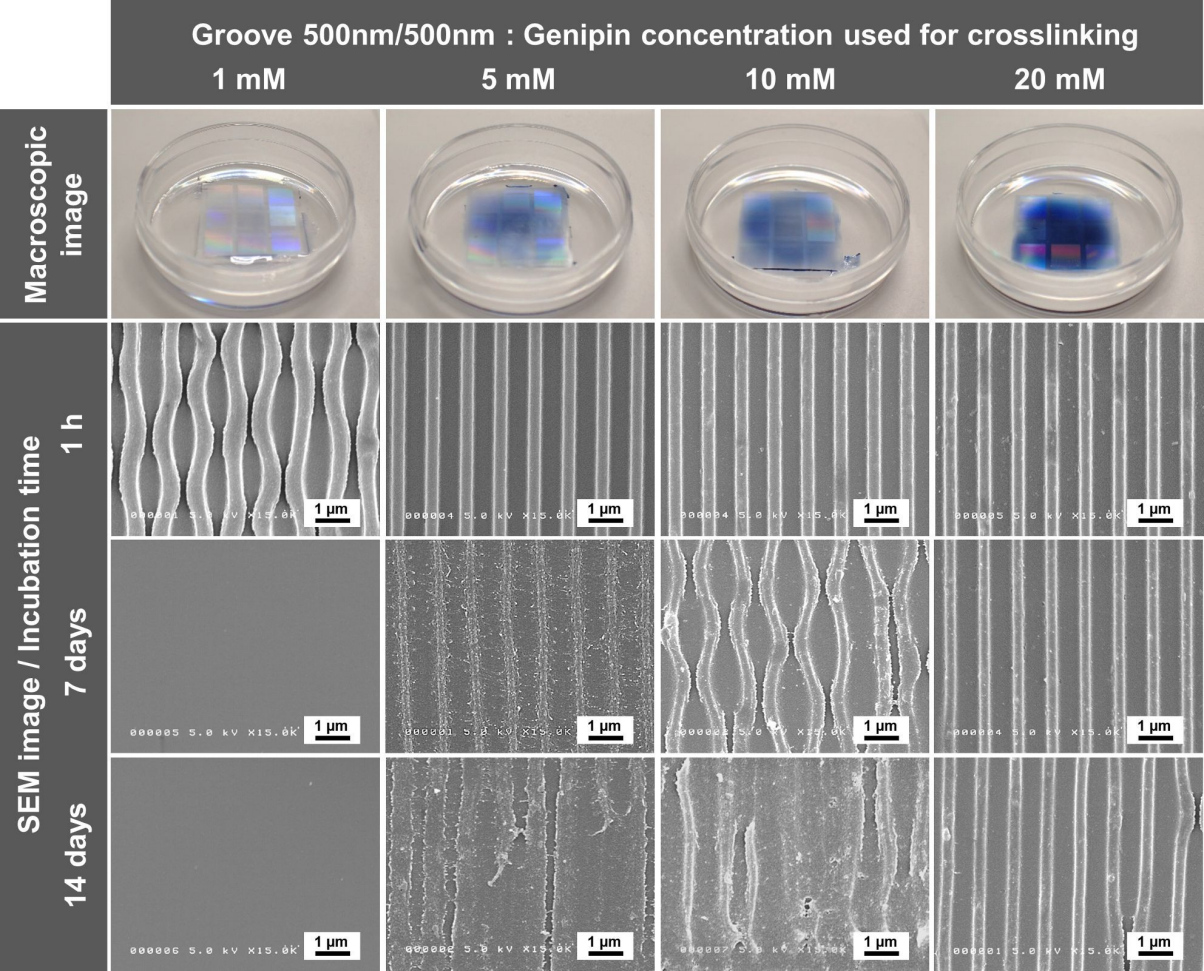
The effect of the addition of genipin at 1, 5, 10, and 20 mM to 20 wt % gelatin on the surface morphology of the gelatin grooves molded to have a width of 500 nm and a height of 500 nm. Upper row: macroscopic images of the different gelatin patterns. Middle and lower rows: SEM images of the surface of the dried gelatin patterns after 1 h, 7 days, and 14 days of incubation in cell culture medium.

Next, we investigated the effect of genipin concentration on the stability of the different gelatin surface patterns in cell culture medium, as shown in Figure 2. The color of the crosslinked patterns gradually became a darker blue as the concentration of genipin increased from 1 to 20 mM. As shown in Table 1, the crosslinking degree of gelatin patterns increased with increasing genipin concentration. The crosslinking degree was obtained over a range of approximately 7–41%. The maximum crosslinking degree (41%) was obtained at the highest concentration of genipin (20 mM). To understand the stability in the cell culture medium, gelatin surfaces with grooves (500 nm width and 500 nm height) were immersed in Dulbecco’s modified Eagle’s medium (DMEM) containing 10% fetal bovine serum (FBS). After 1 h, 7 days, or 14 days of incubation, the immersed gelatin grooves were fixed with glutaraldehyde and then observed by SEM. The SEM images revealed large differences in the stability of the gelatin grooves, depending on the genipin concentration. After 1 h of immersion in the cell culture medium, the gelatin grooves that were crosslinked with 1 mM genipin swelled and adopted a wave-like appearance. In contrast, the gelatin grooves crosslinked with 5 to 20 mM genipin were generally unaffected by 1 h of immersion in cell culture medium. After 7 days of immersion, gelatin grooves crosslinked with 1 mM genipin were completely dissolved. Similarly, most of the gelatin grooves crosslinked with 5 mM genipin were also dissolved, although the shape of the groove could still be discerned. The gelatin grooves crosslinked with 10 mM genipin became swollen and adopted a wave-like appearance. In contrast, gelatin grooves crosslinked with 20 mM genipin remained relatively unaffected by the 7 day incubation in cell culture medium. At 14 days of immersion, the gelatin grooves crosslinked with 5 or 10 mM genipin showed only a faint outline of the groove. In contrast, the gelatin grooves crosslinked with 20 mM genipin maintained a clear groove shape.

**Table 1:** Crosslinking degree of the gelatin patterns.

Genipin (mM)	Crosslinking degree (%)

1	7 ± 3
5	19 ± 4
10	26 ± 4
20	41 ± 3

**Figure 3:**
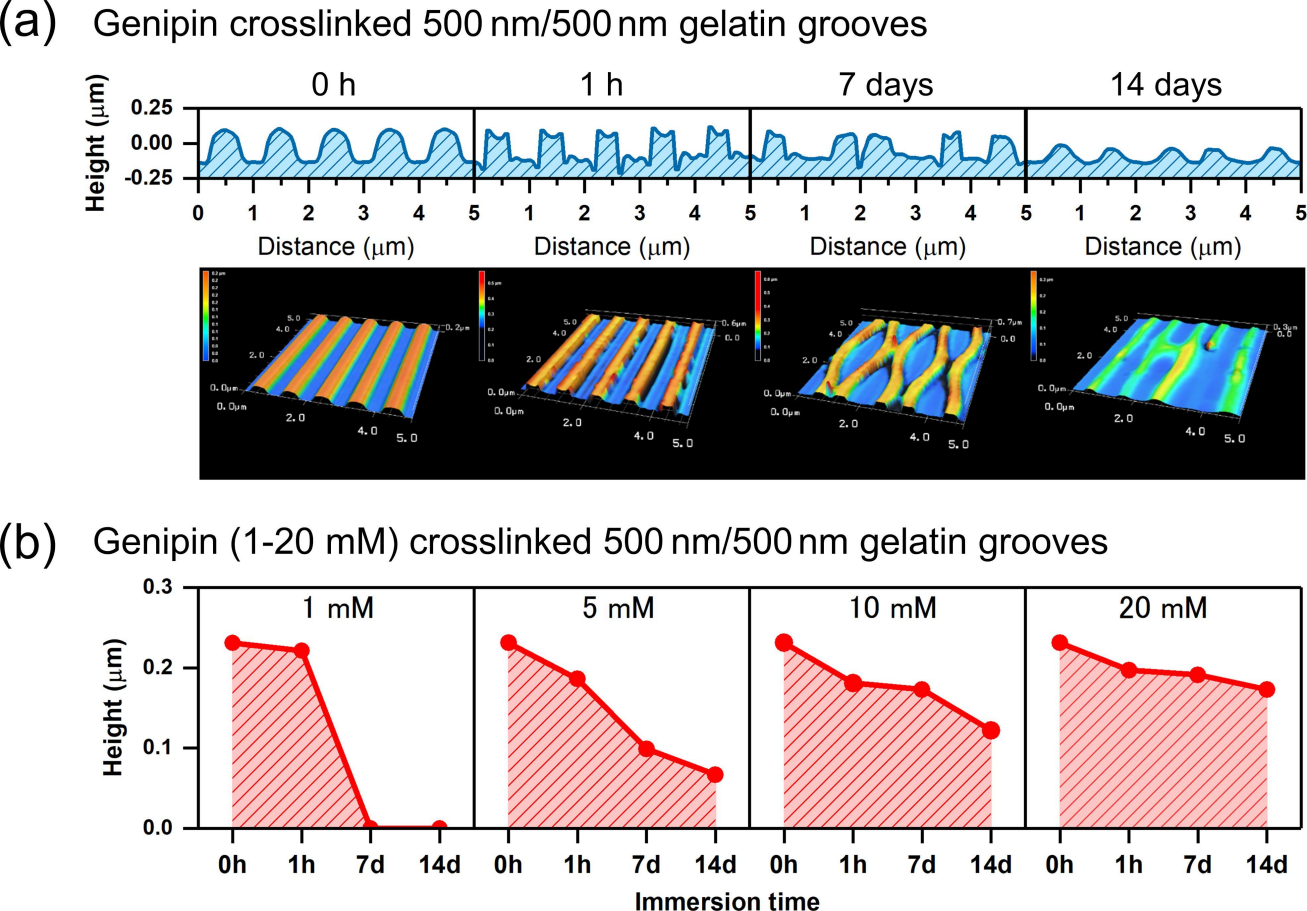
Height changes in gelatin grooves molded to have a width of 500 nm and a height of 500 nm after immersion in cell culture medium. The upper images are (a) laser microscope (LM) images of a groove crosslinked with 10 mM genipin. Immersion times were 0, 1 h, 7 days, and 14 days. The lower graphs show (b) the height of the grooves crosslinked with different concentrations of genipin (1 mM, 5 mM, 10 mM, and 20 mM) over time. The heights of the grooves were estimated by examining the cross-sectional profile of the LM images.

Figure 3a shows typical height changes in gelatin-molded grooves following immersion in cell culture medium over time. In this example, the gelatin grooves were crosslinked with 10 mM genipin and molded to have a 500 nm width and a 500 nm height. Cross-sectional analysis and the surface topological images indicate that the height of the grooves decreased with increasing immersion time. Figure 3b shows the height change over time in the grooves (also molded to be 500 mm wide and 500 nm high) crosslinked with a range of genipin concentrations (1 to 20 mM) and then immersed in cell culture medium. The height of the original grooves was 230 nm before immersion in the cell culture medium. The magnitude of the decrease in groove height depended on the concentration of genipin used for crosslinking. The heights of the grooves after 14 days of immersion in cell culture medium were 0, 70, 120, and 170 nm for 1, 5, 10, and 20 mM genipin, respectively. The smallest height change was observed at the highest concentration of genipin (20 mM). Because of its high stability in cell culture medium, we used gelatin patterned using 20 mM genipin as a crosslinker to subsequently examine cell attachment and proliferation.

### Live/dead cell viability assay of Saos-2 cells on gelatin patterns

**Figure 4:**
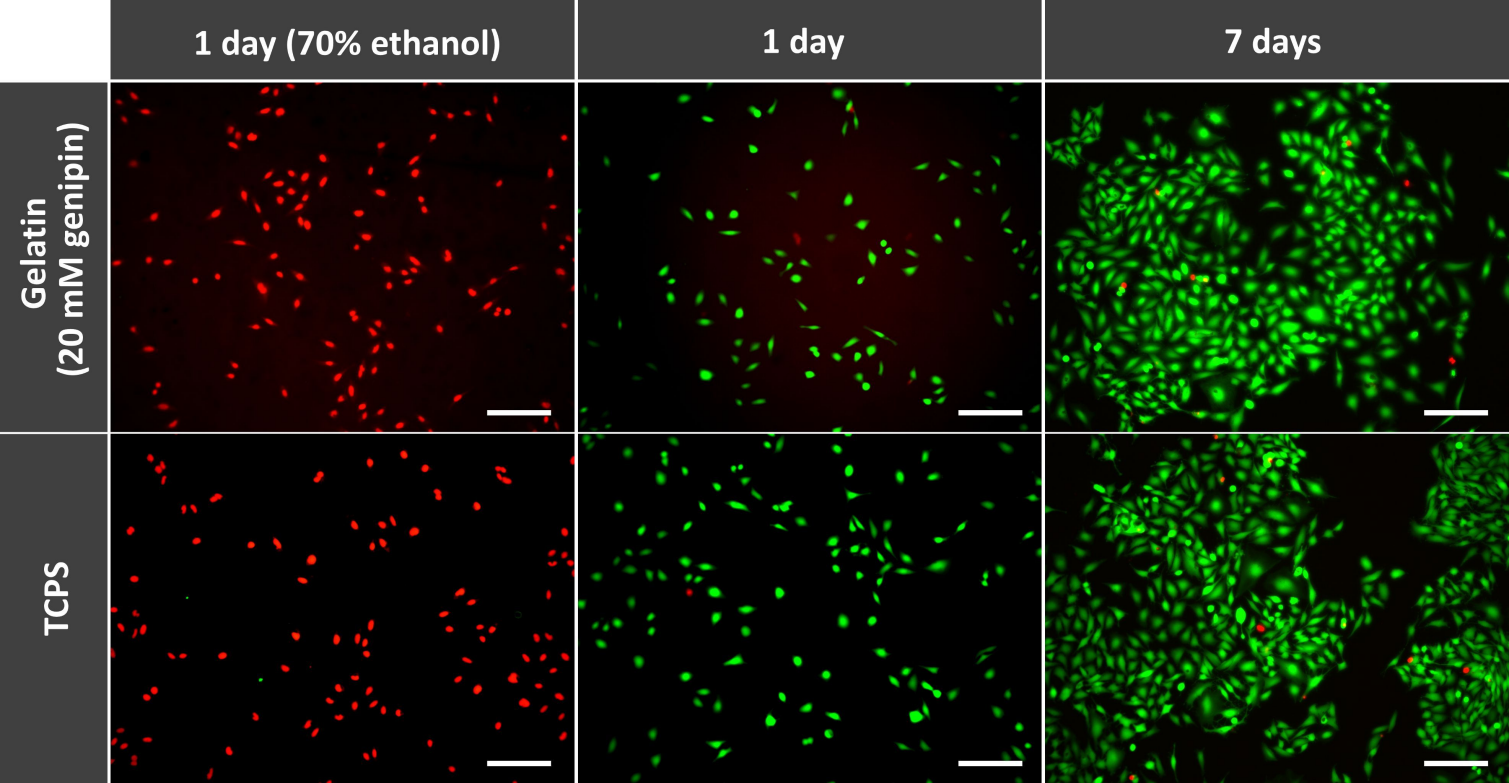
Live/dead cell viability assay of Saos-2 cells cultured on the gelatin crosslinked with genipin. The gelatin surface, with a diameter of 500 nm and a height of 500 nm and crosslinked with 20 mM genipin, was used as a pattern, and tissue cultured polystyrene (TCPS) was used as a control. The cells were cultured for 1 day or 7 days and then stained with the Cellstain double staining kit. The live and dead cells exhibited green and red fluorescence, respectively. Dead cells pretreated with 70% ethanol for 30 min were used as controls for dead cells. The scale bar represents 100 µm.

The cell viability of Saos-2 cells on the gelatin crosslinked with 20 mM genipin was easily estimated with live/dead double staining (Figure 4). The gelatin pillars, with diameters of 500 nm and heights of 500 nm, were used as gelatin patterns. The high ratio of green-stained cells on gelatin, which indicated the presence of live cells on the crosslinked gelatin, was similar to the high ratio of green-stained cells on a tissue cultured polystyrene (TCPS), which was used as a control for a high-viability surface after 1 day or 7 days of culturing. The number of red-stained cells was few, indicating that dead cells were observed on both surfaces. Dead cells pretreated with 70% ethanol were used as controls for dead cells; all the cells on both surfaces were observed as red-stained cells. Crosslinking with 20 mM genipin did not affect the viability of the cells on the gelatin pattern.

### Morphology of Saos-2 cells attached to gelatin patterns after 1 h incubation

**Figure 5:**
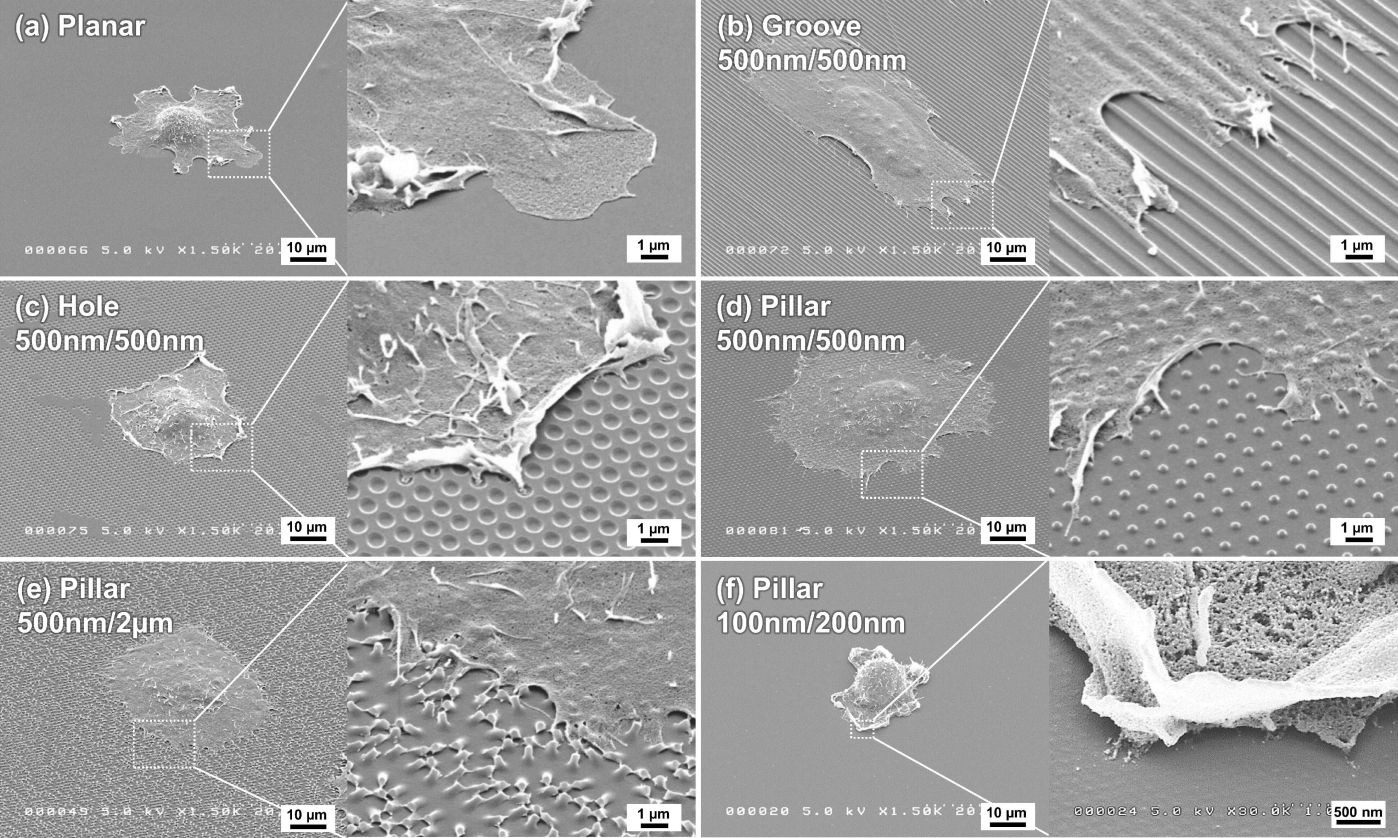
SEM images of attached Saos-2 cells on different gelatin patterns after 1 h incubation at a 45 °C tilt angle. The patterned surfaces were (a) a planar surface, (b) grooves, (c) holes, and (d) pillars molded to have a diameter of 500 nm and a height of 500 nm. (e) Pillars molded to have a diameter of 500 nm diameter and a height of 2 µm. (f) Pillars molded to have a diameter of 100 nm diameter and a height of 200 nm. The indicated sizes are the pattern sizes of the mold.

The typical morphology of Saos-2 cells attached to the different gelatin patterns is shown in Figure 5. Cells attached on a planar surface, holes, or pillars exhibited radial spreading, especially the cells attached on pillars with a diameter of 500 nm (Figure 5d and Figure 5e). Cells attached on pillars or holes with diameters of 500 nm exhibited randomly elongated filopodia and lamellipodia spreading from the cell body. The filopodia of the cells seemed to attach to the pillar, or penetrate into the hole. In particular, the filopodia of cells attached on pillars with a 2 µm height seemed to be particularly well-developed (Figure 5e). Cells attached on the groove assumed a spindle-like shape that was formed along the direction of the groove (Figure 5b). In this case, cell filopodia appeared to be attached to the ridge of the groove. In contrast, cells attached on pillars with a 100 nm diameter had difficulty spreading (Figure 5f). It was also noted that cells attached on a 100 nm pillar, or attached on a planar surface, extended a much smaller number of filopodia, compared to the cells attached on grooves, holes, or pillars with a width or diameters of 500 nm.

### Immunofluorescence staining after 1 day of culturing

**Figure 6:**
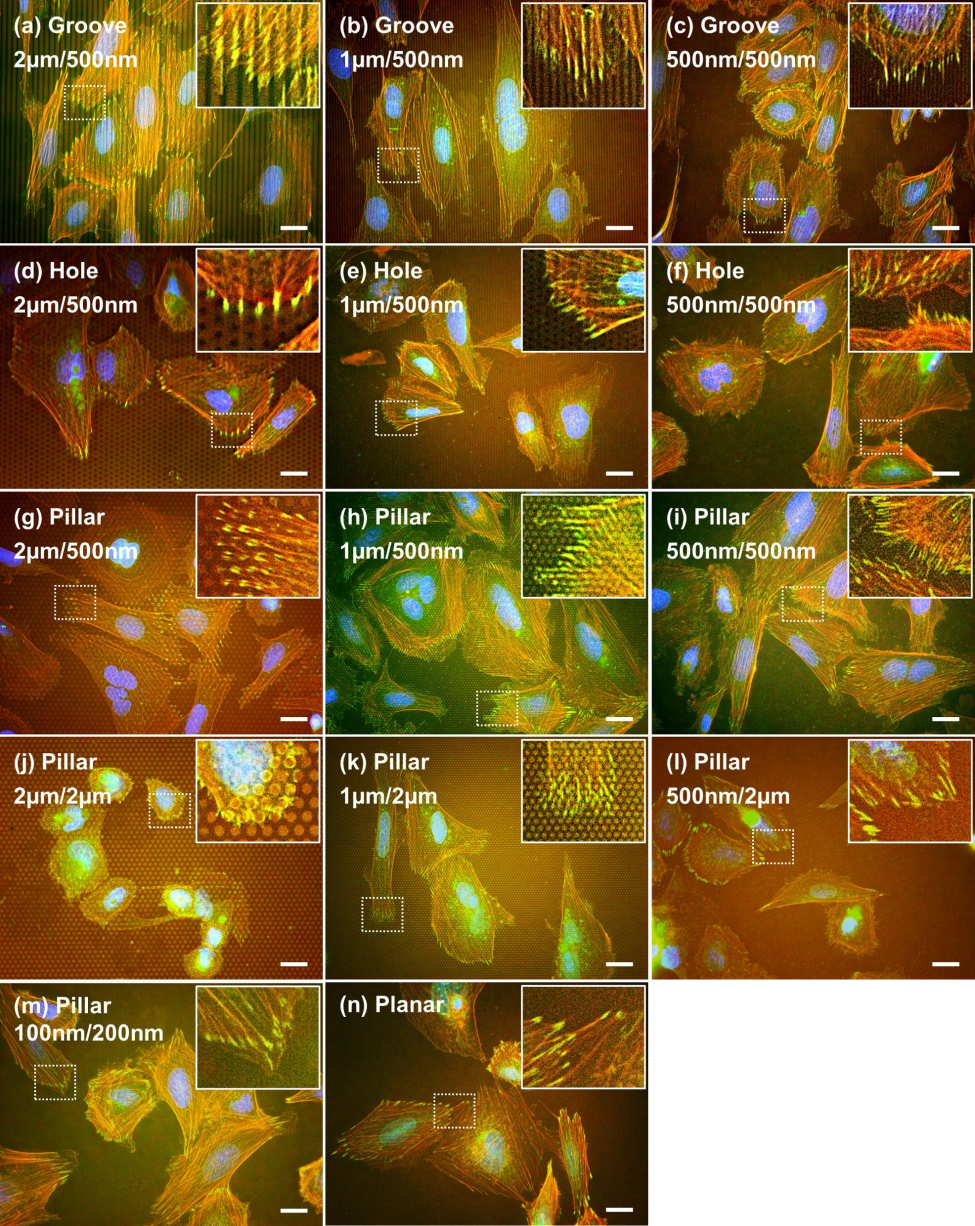
Immunofluorescence images of Saos-2 cells on the gelatin patterns after 1 day of culturing. The cells were on patterns of (diameter or width/height or depth) grooves with (a) 2 µm/500 nm, (b) 1 µm/500 nm, and (c) 500 nm/500 nm; holes with (d) 2 µm/500 nm, (e) 1 µm/500 nm, and (f) 500 nm/500 nm; pillars with (g) 2 µm/500 nm, (h) 1 µm/500 nm, (i) 500 nm/500 nm, (j) 2 µm/2 µm, (k) 1 µm/2 µm, (l) 500 nm/2 µm, (m) 100 nm/200 nm; and (n) planar. Typical images of each pattern surface are presented. Red = F-actin; green = vinculin; blue = nuclei. Inset was magnified from dotted squares in each pattern. Scale bar represents 20 µm.

Immunofluorescence images of Saos-2 cells on the gelatin patterns are shown in Figure 6. Vinculin (green), F-actin (red), and nuclei (blue) of the cells were stained after 1 day of culturing. The cells were stained after 1 day of culturing because vinculin and actin were not fully expressed after 1 h of incubation. The shape and size of the gelatin patterns used were as follows: Grooves and holes, that were molded to be 2 µm, 1 µm, or 500 nm in width or diameter, and 500 nm in height, pillars that were molded to be 500 nm in diameter and 2 µm or 500 nm in height, or 100 nm in diameter and 200 nm in height, with the planar surface being used as the control. The fluorescence images revealed that the distribution of vinculin was largely different, depending on the shape and size of the pattern surface. Unfortunately, auto-fluorescence, caused by azo-compounds in the crosslinked genipin, slightly interfered with the observation of green and red fluorescence of the cells [50]. The red fluorescence of actin was particular not clear in this observation. The cells and nuclei on the grooves were aligned along the direction of the grooves (Figure 6a–c). The vinculin in the cells on the grooves was observed on the top of the ridge and was aligned along the direction and size of the ridge. The orientation of the cells and nuclei on the grooves with widths of 500 nm and heights of 500 nm was weaker than that of the cells and nuclei on the grooves with widths of 2 µm or 1 µm and heights of 500 nm. The vinculin in the cells on the holes was observed on the upper surface, rather than the inner depth of the hole, but the distribution of vinculin on the holes with diameters of 500 nm and depths of 500 nm was not clear (Figure 6d–f). Interestingly, vinculin on pillars with larger diameters was observed on the top or the top edge of the pillars (Figure 6g,h,j,k). Distribution of vinculin on pillars with smaller diameters did not show any clear association with the pillar shape (Figure 6i,l,m). Furthermore, a large number of small spots were observed on pillars with diameters of 1 µm or 500 nm and heights of 500 nm. The number and size of vinculin spots on pillars with diameters of 100 nm and heights of 200 nm was similar to the number and size of vinculin spots on the planar surface (Figure 6m,n).

### Cell attachment to different gelatin patterns after 1 h of incubation

**Figure 7:**
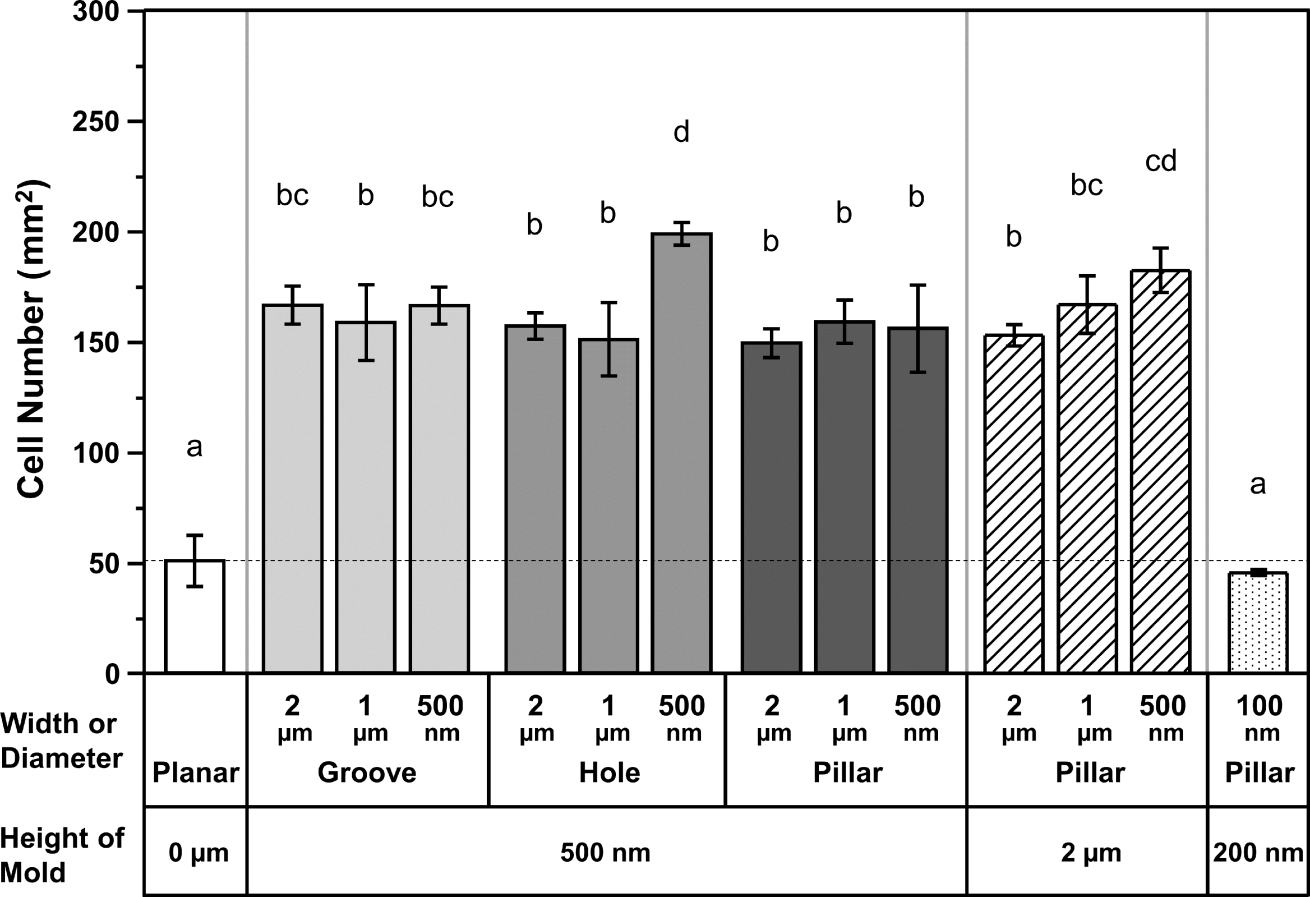
Cell attachment of Saos-2 cells on different gelatin patterned surfaces. Saos-2 cells were incubated for 1 h with the different gelatin patterned surfaces and the number of attached cells/mm^2^ was determined by counting the number of cells visible in microscope images (*n* = 6). The dotted line indicates the average cell number observed on the planar surface. The indicated sizes are the pattern sizes of the mold. Significant differences among the groups, measured with the Tukey’s multiple comparison test, are indicated above each column with different letters (*p* < 0.05). In other words, columns with the same letter are not significantly different.

To estimate the effect of shape and size of the different gelatin patterns on cell attachment, we carried out a cell attachment assay using Saos-2 cells incubated for 1 h with the different patterned gelatin matrices. A short incubation time of 1 h was adopted to evaluate the initial attachment ability; this is because faster cell attachment to the surface of dental implants, considered as one of the applications of gelatin patterns, is important to avoid bacterial contamination from the oral cavity. As shown in Figure 7, the number of attached cells on the different gelatin patterns was significantly higher than on the planar gelatin surface. The average number of cells on the gelatin patterns, with the exception of the pillar with a diameter of 100 nm, were approximately 3 to 4 times higher than on the planar surface. The highest number of attached cells was observed on holes with a diameter of 500 nm (*p* < 0.05). The second-highest number of cells was observed on pillars with a diameter of 500 nm and a height of 2 µm. The lowest number of cells was observed on pillars with a diameter of 100 nm, which was similar to the number of cells found on the planar surface. However, there was no significant difference in the number of attached cells among most of the gelatin patterns, with the exception of the hole with a diameter of 500 nm, the pillar with a width of 500 nm and height of 2 µm, and the pillar with a diameter of 100 nm (*p* > 0.05).

### Morphology of Saos-2 cells cultured on the different gelatin patterns after 7 days of incubation

**Figure 8:**
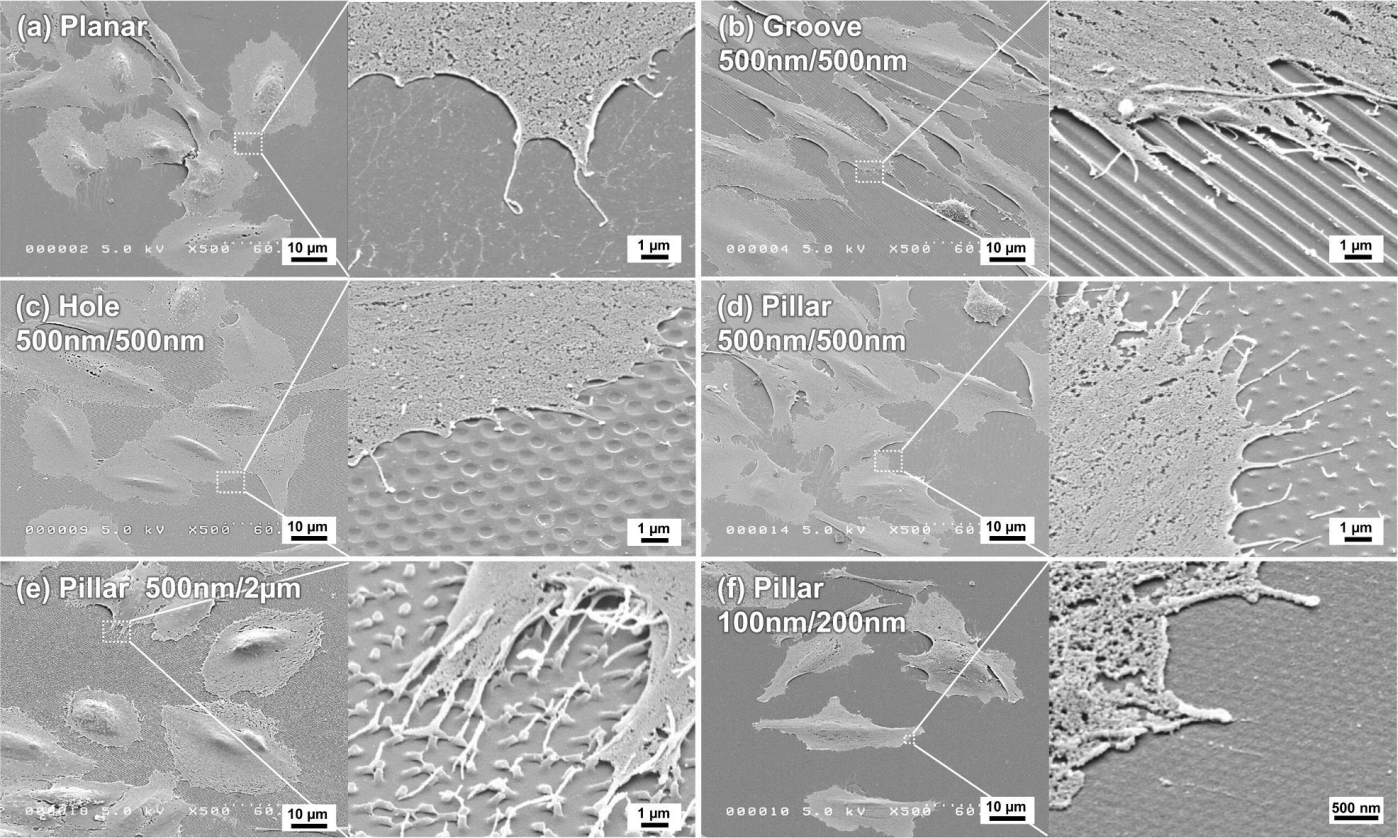
SEM images of the attached Saos-2 cells on the different gelatin patterns after 7 days of incubation at a 45 °C tilt angle. The patterned surfaces were (a) a planar surface, (b) grooves, (c) holes, (d) pillars molded to have a diameter of 500 nm and height of 500 nm, (e) pillars molded to have a diameter of 500 nm and a height of 2 µm, and (f) pillars molded to have a diameter of 100 nm diameter and a height of 200 nm. The indicated sizes are the pattern sizes of the mold.

The typical morphology of Saos-2 cells after 7 days of culture on the different gelatin surface patterns is shown in Figure 8. As expected from the previous experiments, the shape of the gelatin surface patterns crosslinked with 20 mM genipin remained mostly stable after 7 days of cell culture, although the shape of the gelatin surface patterns was slightly smoother than after 1 h of incubation. The pillar with a diameter of 500 nm and a height of 2 µm height collapsed because of its softness (Figure 8e). In general, the morphology of the cells grown on the different gelatin surface patterns after 7 days of incubation was similar to that of the cells seen at 1 h of incubation. Cells cultured on the planar surface, the holes, and the pillars were found to spread radially. On the other hand, most of the cells attached on the groove were aligned along the direction of the groove (Figure 8b). While numerous filopodia were observed in cells grown on pillars with diameters of 500 nm, only a small number of filopodia were noted for cells grown on pillars with diameters of 100 nm, or on the planar surface.

### Immunofluorescence staining after 7 days of culturing

**Figure 9:**
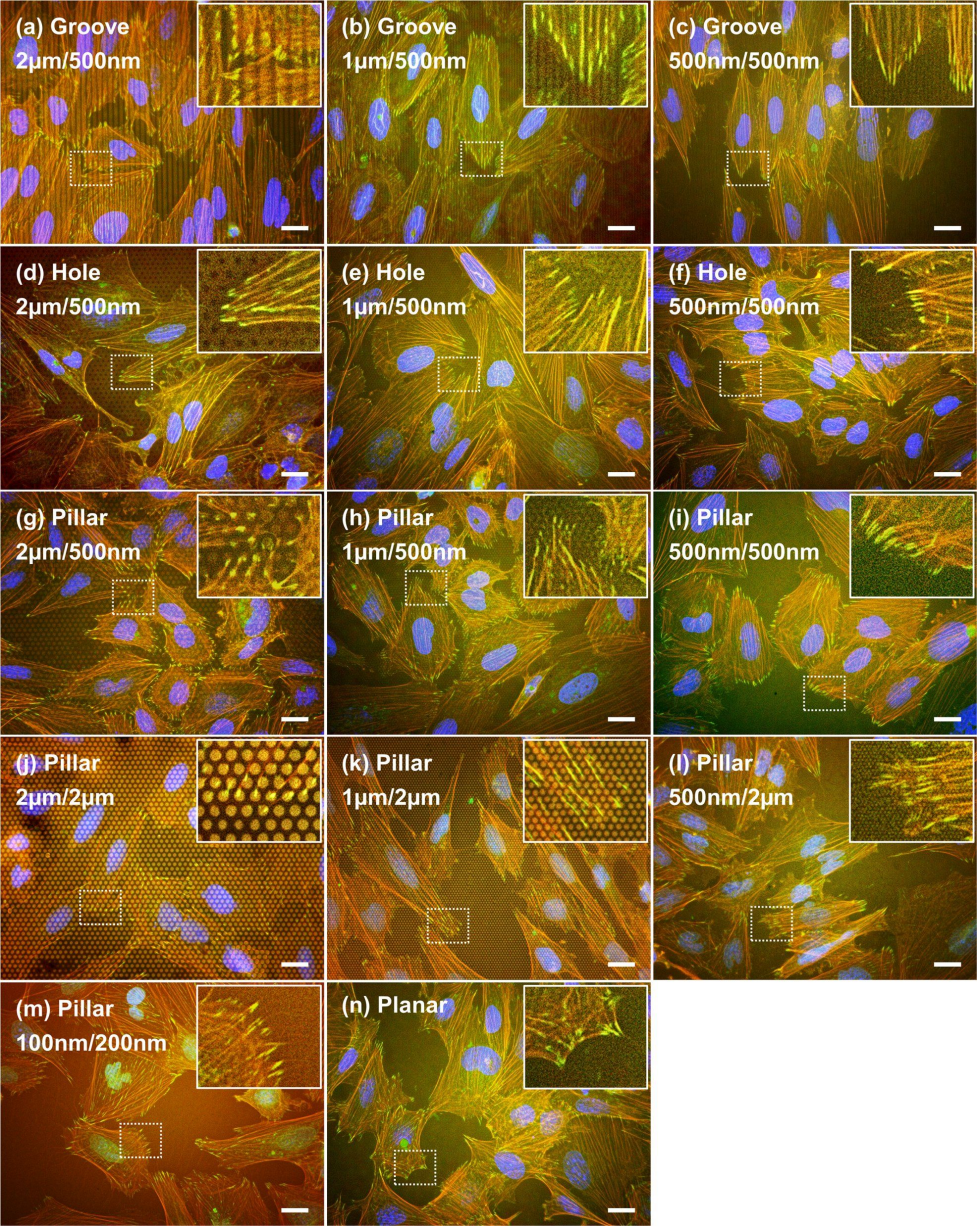
Immunofluorescence images of Saos-2 cells on gelatin patterns after 7 days of culturing. The cells were on the following patterns (diameter or width/height or depth): grooves with (a) 2 µm/500 nm, (b) 1 µm/500 nm, and (c) 500 nm/500 nm; holes with (d) 2 µm/500 nm, (e) 1 µm/500 nm, and (f) 500 nm/500 nm; pillars with (g) 2 µm/500 nm, (h) 1 µm/500 nm, (i) 500 nm/500 nm, (j) 2 µm/2 µm, (k) 1 µm/2 µm, (l) 500 nm/2 µm, (m) 100 nm/200 nm; and (n) planar. Typical images of each pattern are presented. Red = F-actin; green = vinculin; blue = nuclei. Inset was magnified from dotted squares in each pattern. The scale bar represents 20 µm.

Immunofluorescence images of Saos-2 cells on the gelatin patterns after 7 days of culturing are shown in Figure 9. The images of vinculin (green), F-actin (red), and nuclei (blue) were quite similar to the images obtained after 1 day of culturing (Figure 6). Briefly, the cells and nuclei on the grooves were aligned along the direction of the grooves. The vinculin in the cells on each pattern was observed on the top or top edge of a ridge/groove (Figure 9a–c), the upper surface (rather than the inner depth of a hole (Figure 9d and Figure 9e)), and the top or top edge of a pillar (Figure 9g,h,j,k). However, the distribution of vinculin on the holes and the pillars with diameters of 500 nm and depths of 500 nm was not clear (Figure 9f,i). The number and size of vinculin spots on pillars with diameters of 100 nm and heights of 200 nm was similar to the number and size of vinculin spots on the planar surface (Figure 9m,n). Nevertheless, a slight difference was observed; the fluorescence strength of vinculin on the pillars with heights of 2 µm (Figure 9j,k,l) was slightly weaker than the fluorescent strength after 1 day of culturing.

### Cell proliferation assay of Saos-2 cells grown on the different gelatin patterns after 7 days of incubation

**Figure 10:**
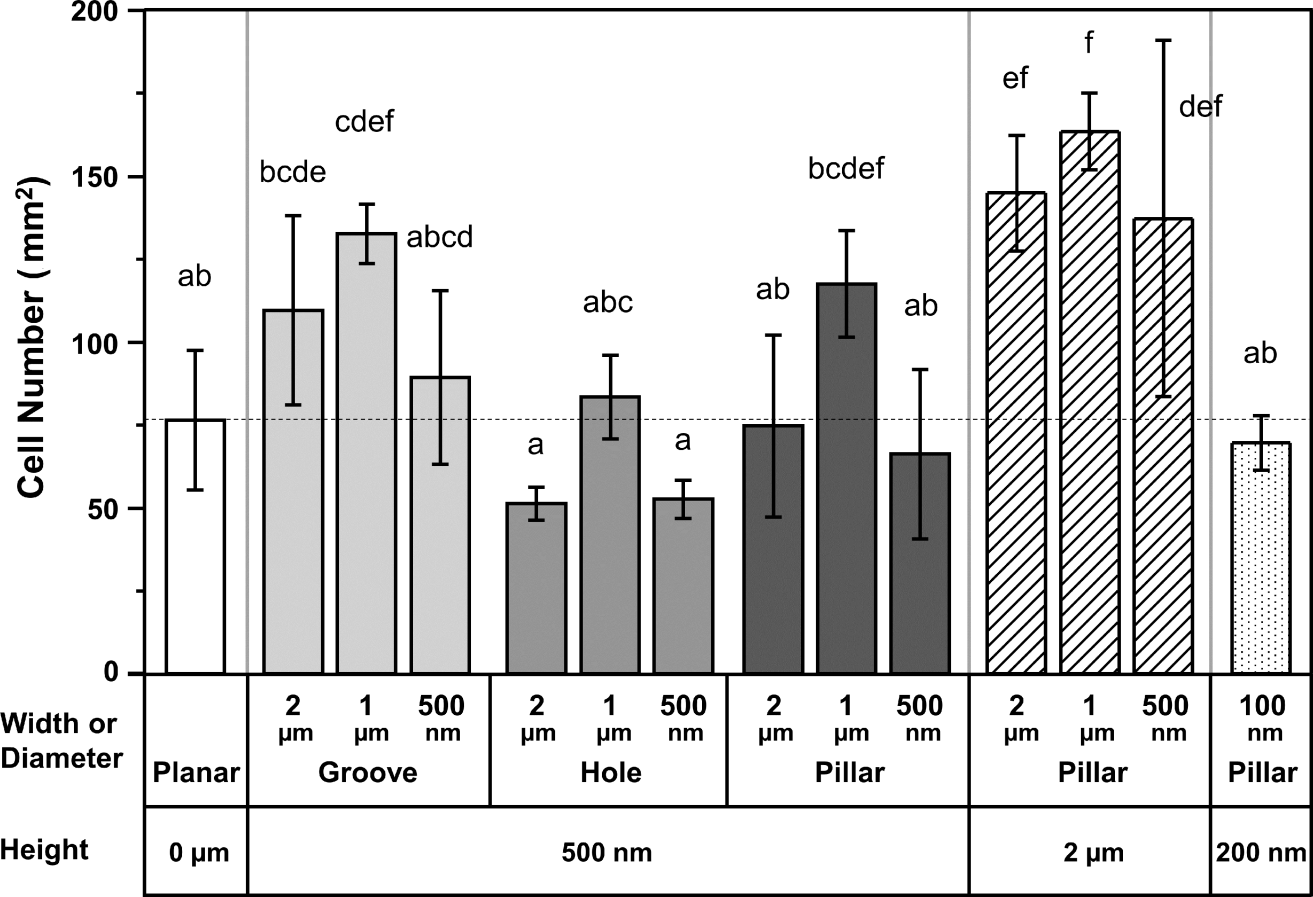
Cell proliferation assay of Saos-2 cells on the different gelatin patterns. Saos-2 cells were incubated for 7 days of the indicated gelatin surface patterns (*n* = 6). The dotted line indicates the average cell number on the planar surface. The indicated sizes are the pattern sizes of the mold. Significant differences among the groups, measured with the Tukey’s multiple comparison test, are indicated above each column with different letters (*p* < 0.05). In other words, columns with the same letter are not significantly different.

Figure 10 shows the number of Saos-2 cells on the different gelatin surface patterns after 7 days of culture. The trend in the cell proliferation assay was different from the cell attachment assay (Figure 7). The highest cell number was observed on pillars with diameters of 1 μm and heights of 2 µm. The average number of cells on these pillars were approximately 2.1 times higher than average number cells on the planar surface. Interestingly, the number of cells on the grooves, holes, and pillars with a width or diameter of 1 µm was the highest among the same shape and height gelatin groups. While the number of cells on the grooved surface was higher than in the other surface shape groups having a height of 500 nm, the number of cells on the surface having holes, was lower than on surfaces with patterns having a 500 nm height.

## Discussion

### Preparation of gelatin patterns

In this study, we created gelatin surfaces with micro- to nanoscaled patterns using a mold and crosslinking with genipin, a low toxicity natural product. Gelatin or collagen patterning with genipin has been referred to in previously studies, but not as a main focus [38–39,51–52]. The crosslinking reaction of gelatin is controlled by the reaction time, the temperature, and humidity. In this study, we used 20 wt % gelatin as an optimal concentration for the gelatin solution, because a concentration of 30 wt % gelatin produced a solution that had too high a viscosity, making the molding process more difficult. A reaction temperature of 37 °C and a reaction time of 3 days have previously been described as being suitable conditions [51–52]. As required, the mechanical properties of crosslinked gelatins can be changed by altering the crosslinking conditions, such as the concentration of the crosslinker, reaction time, and temperature. Polydimethylsiloxane (PDMS) has the known characteristics of being gas and vapor permeable [53–54]. Therefore, we used a thin PDMS mold, with a thickness of 0.5 mm, to allow water to evaporate through the mold into the air by diffusion during the drying process. This approach is one of the most important aspects of the patterning procedure reported here.

Gelatin patterning of grooves, holes, and pillars 500 nm in width or diameter were fabricated using crosslinking with genipin. We found that the color of the patterned gelatin became darker blue (Figure 2), in agreement with the color of collagen scaffolds crosslinked with genipin [51]. The blue color is derived from a molecular structural change in genipin as it crosslinks either collagen or gelatin. Thus, the darker the blue color, the higher degree of crosslinking with genipin was achieved. Of note, it was not possible to pattern gelatin on the 100 nm diameter scale using this current method (Figure 1f). In previous studies we have demonstrated that on the 500 nm diameter scale, using the impression of hydrocolloid materials such as alginate and agar, it was difficult to fabricate surfaces of sufficient shape and size [14]. Because these colloidal solutions have a high water content they become very condensed during the gelling and drying processes. In this study, it is likely that a gelatin solution in the hole mold required to fabricate the 100 nm pillar shape would become easily shrunken, or could easily escape from the mold during gelation or crosslinking. Thus, gelatin patterning of 100 nm pillars could not be easily achieved.

High-resolution patterning with collagen/gelatin has already been reported [36,45–46]. Collagen grooves, 333 to 650 nm wide, have been successfully fabricated using crosslinking with carbodiimide [46]. Gelatin pillars with a diameter of 250 nm to 1 µm have also been patterned using polymerization with GelMA. The swelling ratio of these pillars could be successfully controlled by changing the GelMA content [45]. Nanogrooves and nanopillars, comprised of gelatin, have also been embossed and crosslinked using formaldehyde. The nanogrooves produced had a width of about 200 nm and a depth of 18 nm, whereas the nanopillars had a diameter of about 2 µm and a height of about 53 nm, respectively [36]. Since the submicro- to nanoscale of our gelatin patterning method is similar to the scale of these high-resolution patterning methods, our method could be a candidate for future high-resolution patterning of gelatin. Furthermore, genipin crosslinking has the advantages of being commercially available, of low toxicity, and biodegradable.

### Stability of the different gelatin patterns in cell culture medium

A major finding from this study is that the stability of the different gelatin patterns in cell culture medium can be controlled by the degree of genipin crosslinking. In this preliminary study, our target for minimum crosslinking degree was to maintain a fine pattern shape in the cell culture medium for at least 14 days. Excessively high crosslinking degree could alter the gelatin properties that are favorable for cell culture. Fourteen days of culture will be the minimum period required to estimate bone-related functions of osteoblasts in the future. Swelling and degradation of the different gelatin patterns examined here in the presence of cell culture medium were largely influenced by the concentration of genipin. In general, gelatin patterns with a lower degree of crosslinking had a higher swelling ratio, and a faster degradation than patterns with a higher degree of crosslinking. The gelatin patterns examined here also showed a similar tendency to solubilize more quickly at low concentrations of the genipin crosslinker, as shown in Figure 2. The high stability of our gelatin patterns, crosslinked using 20 mM genipin that resulted in a crosslinking degree of 41%, was similar to the stability of collagen grooves crosslinked with carbodiimide [46]. In our study, the collagen grooves retained their structure for at least 14 days in cell culture medium, suggesting that the solubility and degradability of the different patterns can be controlled by the concentration of genipin used for crosslinking.

One limitation of this study is that the actual size of the different gelatin surface patterns could not be determined in cell culture due to technical reasons related to the presence of cell culture medium. It is possible that the different gelatin patterns might swell more in cell culture medium than the dry state of gelatin. To determine the accurate size of the gelatin patterns in cell culture medium [45], it may be effective to measure the size change of swelling gelatin patterns with immersion time in the cell culture medium by using atomic force microscopy or high-resolution confocal three-dimensional microscopy in liquid. Another limitation is that optimized reaction conditions have not been thoroughly investigated with conditions such as longer reaction time, higher or lower reaction temperature, pH, and higher genipin concentration. Nevertheless, the crosslinking by genipin has the potential to control the solubility, degradability, and softness of gelatin patterns. In the future, we plan to examine the behavior of cells on soft and hard gelatin surface patterns, crosslinked with genipin in the range of 1 to 40 mM.

### Morphology of Saos-2 cells on the different gelatin patterns after 1 h and 7 days of incubation

Cell morphology was largely affected by the shape and size of the different gelatin patterns. Our data showing that osteoblastic Saos-2 cells orientate in the direction of gelatin grooves is in agreement with data showing the orientation of osteoblasts in gelatin grooves [55] and osteoprogenitor cells in collagen grooves [44]. In the above studies, well-aligned osteoblast cells were reported to be present in grooves with widths between 500 nm and 27 µm, using either gelatin or collagen substrates. However, grooves with a width of 100 µm width did not influence osteoblast cell orientation [44]. Good cell alignment in collagen or gelatin grooves has also been reported for NIH-3T3 cells [36], vascular smooth muscle cells [46], and dental pulp stem cells [56]. In several types of cells, the grooves with widths ranging from submicrometers to several tens of micrometers can effectively maneuver to align the cells [57–58]. However, Zorlutuna et al. have reported that human microvascular endothelial cells were not aligned in collagen grooves with widths of 333, 500, or 650 nm. Additionally, Vernon et al. have reported that the depth factor of the groove also has an influence on cell orientation [34]. In this regard, human dermal fibroblasts, as well as human umbilical artery smooth muscle cells, have been reported to be not well-aligned in grooves with depths of 200 or 560 nm, but were well-aligned in grooves with a depth of 2 µm. With respect to the grooves used in our study, it appeared that a gelatin groove depth of 500 nm was sufficient for alignment of Saos-2 cells. Therefore, grooves with widths of 500 nm to 2 µm and a 500-nm depth are suitable for the alignment of Saos-2 cells.

Saos-2 cells cultured on certain gelatin pillars developed filopodia that elongated radially from the Saos-2 cell body. Cells on pillars with a diameter of 500 nm and height of 500 nm (Figure 5d) or 2 µm (Figure 5e) exhibited larger cell areas and numerous filopodia after 1 h of incubation. In contrast, cells on pillars with a diameter of 100 nm and a height of 200 nm had a smaller cell area and fewer filopodia. Thus, the height of the pillar is an important factor in controlling cell behavior. We also observed the attachment of several filopodia tips to the top of the pillars, as previously reported [59]. In a previous study, Saos-2 cells on poly (lactic acid) (PLLA) pillars with diameter of 200 nm and height of 900 nm extended numerous filopodia radially and the tips of the filopodia were attached to the top of the pillars [59]. However, the small cell area seen on PLLA pillars was not in agreement with the large cell area seen on our gelatin pillars. PLLA pillar surfaces are hydrophobic because their contact angle is about 85°, whereas in contrast, our gelatin pillars are highly hydrophilic. For this reason, cells grown on gelatin pillars are more likely to generate a larger cell area. Cells cultured on gelatin holes also spread radially. Although their filopodia seemed to be inserted into holes after 1 h of incubation (Figure 5c), the filopodia were found to be attached to the upper surface surrounding the hole after 7 days of incubation (Figure 8c). The filopodia attached to the upper surface surrounding the hole were also observed with immunofluorescence microscopy (Figure 9d and Figure 9e). It therefore appears that Saos-2 cells prefer the upper surface of gelatin holes rather than the interior of the hole. The shape of the cell body and the number of filopodia on Saos2 cells grown on a surface containing gelatin holes was similar to cells grown on a planar gelatin surface.

The interaction between focal adhesions containing vinculin and biomaterials was important to understanding the behavior of cell attachment or a related signal transduction [60]. Thus, vinculin, a major component of focal adhesions, was stained to estimate the direct connection between the interface of cells and gelatin patterns. The distribution of vinculin was largely affected by the size and shape of gelatin patterns (Figure 6 and Figure 9). The vinculin in the cells on large-sized patterns was observed on the top or top edge of a ridge/groove [61], the upper surface, rather than the inner depth of a hole, and the top or top edge of a pillar [62] after 1 day and 7 days of culturing. The regulated distribution of vinculin spots was in agreement with the distribution of vinculin in Saos-2 cells on titanium-coated micro/nanopatterns [13]. However, the distribution of vinculin on the gelatin pillar with a diameter of 500 nm and a height of 500 nm was not clear more than the regulated distribution of vinculin on the same-sized titanium-coated pillar [13]. Furthermore, the strength of vinculin spots on pillars with heights of 2 µm after 7 days of culturing appeared weaker than the strength after 1 day of culturing. The difference in distribution or strength of vinculin spots may be caused by substrate stiffness [63].

These data support the concept that patterning of gelatin shapes, using genipin crosslinking, can easily fabricate “sharp” patterns that can control the morphology and vinculin expression of cells grown on these surfaces. This control of cell shape and vinculin expression by patterning is known to be able to control cell adhesion, function, and differentiation [60,64–65]. Therefore, a thoughtful design of the shape and size of gelatin patterned surfaces has the potential to control cell function and differentiation.

### Cell attachment and proliferation assay

The data from this study indicate that the number of Saos-2 cells attached to a gelatin surface can be improved by creating micro- or nanopatterns. In addition, cell proliferation was also significantly affected by the size and shape of the patterns on the gelatin surface. Interestingly, the effect of size and shape of the gelatin surface on cell proliferation was different than it was for cell attachment. Moreover, the live/dead cell viability assay demonstrated that gelatin crosslinked with genipin showed low cytotoxicity (Figure 4). The low cytotoxicity for Saos-2 cells on our gelatin patterns was in agreement with the low cytotoxicity for fibroblasts on genipin-crosslinked gelatin [66]. Therefore, genipin-crosslinking could be a viable approach for gelatin patterning.

In general, different gelatin surface patterns improved Saos-2 cell attachment. The number of attached cells on the gelatin surface patterns was approximately 3–4 times higher than that on planar gelatin (Figure 7). This improvement in cell attachment, as a result of the different gelatin surface patterns, was similar to the improvement of cell attachment seen for human osteoblasts and human gingival fibroblasts on gelatin grooves, with a width of 500 nm to 2 µm, created by nanoimprint and thermal crosslinking [55]. In contrast, Chen et al. have reported that after a 4 h incubation time, the number of C2C12 skeletal myoblasts attached to a gelatin micro-groove with a width of 10 µm was lower than the number of cells attached to planar gelatin [42]. These reports suggest that cell attachment behavior to gelatin patterns depends on the cell type. However, there are few reports addressing the attachment of Saos-2 cells to patterned collagen/gelatin surfaces over the 500 nm to 2 µm level. With respect to cell attachment of Saos-2 cells on patterns created on the other materials, many studies have shown an improvement of cell attachment as a result of patterning [11,13,59]. Improvement of Saos-2 cell attachment by our gelatin patterning is similar to the improvement of Saos-2 cell attachment to a pillar with a diameter of 200 nm by PLLA patterning [59]. Our previous data examining Saos-2 cell attachment on apatite surfaces with different patterns shows a similar effect to that seen in this study [11]. This latter study examined the cell attachment of Saos-2 cells on apatite surfaces containing surface patterns with a 2 µm groove, a 2 µm hole, and 500 nm to 2 µm pillars, and showed that cell attachment was largely increased compared to planar apatite. Additionally, Saos-2 cell attachment to titanium surfaces containing 500 nm to 2 µm grooves or pillars showed a similar tendency [13]. Saos-2 cells may therefore prefer materials with an adequately rough surface and moderate wettability.

With regard to the cell attachment of other cell types to other patterned surface, an improvement in cell attachment, due to patterning, depends on several different conditions. An increased tendency to attach to a patterned surface compared to a planar surface has been seen with human dental pulp-derived stem cells on tantalum pillars with diameters of 1 to 6 µm [67]. In contrast, Ca9-22 cells were shown to preferentially attach to a planar titanium surface, rather than one coated with grooves or with pillars with diameters of 500 nm to 2 µm [68]. Interestingly, Özçelik et al. have reported that bone marrow stem cells had a decreased tendency to attach to 200 nm PLLA pillars, compared to the increased attachment tendency of Saos-2 cells [59]. In contrast, C2C12 cells show a different cell attachment tendency on different materials. A surface patterned with a 4.5 µm PDMS pillars increased cell attachment of C2C12 cells, compared to a planar PDMS surface. However, poly(ethylene oxide) or poly(butylene terephthalate) (PEOT/PBT) surfaces, containing the same size pillars as the PLLA pillars, reduced cell attachment of C2C12 cells compared to the corresponding planar surface [69]. The different behavior of cells on patterned surfaces suggests that both cell and material type, in addition to pattern shape and size, are also important factors in determining the degree of cell attachment. Other factors such as wettability, chemical composition, cell seeding density, and softness, also influence the number of attached cells on different patterns [37,59,70–71]. In our case, Saos-2 cells appear to prefer gelatin surface patterns with widths or diameters in the range of 500 nm to 2 µm, rather than planar gelatin. In addition, we speculate that the difference in micro/nanostructures or wettability of gelatin patterns could cause the different attachment behaviors. Of note, the gelatin hole used in this study, with a diameter of 500 nm, exhibited the greatest degree of cell attachment. However, we cannot currently explain the reason behind this. It is possible that cell attachment is improved as a result of increased “softness”, a large upper area, and numerous edges created by the small hole pattern. In the future, it will be necessary to explore the factors responsible for the correlation between cell attachment and patterns in detail.

The different gelatin surface patterns had a large effect on cell proliferation of Saos-2 cells after 7 days of culture (Figure 10). Interestingly, the effect on cell proliferation was different than that on cell attachment (Figure 7). While the number of cells present on holes with a height of 500 nm and a diameter of 2 µm were somewhat lower than the number of cells on the planar surface, the number of cells on grooves with a height of 500 nm height, and pillars with a height of 2 µm, were higher than the number of cells grown on a planar surface. Additionally, the number of cells on the grooves, holes, and pillars with a width or diameter of 1 µm was the highest among the same shape and height gelatin groups. These results support the hypothesis that the size and shape of gelatin surface patterns can have a large influence on cell proliferation. A similar improvement in cell proliferation using patterning has been observed on collagen/gelatin pillars for certain cell types [45–46]. In one study, the cell proliferation of corneal endothelial cells on GelMA pillars with a diameter of 250 nm or 1 µm was found to be increased compared to the planar GelMA surface [45]. Zorlutuna et al. have also shown that cell proliferation of vascular smooth muscle cells in collagen grooves with widths of 333 nm to 600 nm was higher than on planar collagen [46]. On the other hand, it has also been reported that cell proliferation of microvascular endothelial cells on a surface with collagen grooves was similar to that seen on planar collagen [37]. Furthermore, cell proliferation of mouse neuronal C17.2 stem cells on pillars with a diameter of 500 nm or 2 µm with high aspect ratio gelatin-coated polycarbonates was decreased compared to culture on a planar surface [72]. These results using collagen/gelatin patterns demonstrate that cell types and pattern shapes are important factors for cell proliferation on patterned surfaces.

The type of surface material used to create the surface patterns is also an important factor in the proliferation of Saos-2 cells. Saos-2 cell proliferation on polypropylene pillars with a diameter of 20 µm has been reported to be increased compared to proliferation on the planar surface [70], however cell proliferation on surfaces patterned with poly(3-hydroxybutyrate-*co*-3-hydroxyvalerate) (PHBV) or PLLA grooves, with widths of 20 µm, decreased compared to cell proliferation on the respective planar surfaces [71]. On the other hand, cell proliferation on an apatite-coated silicon surface coated with pillars having a diameter of 4 µm was equivalent to that seen on planar apatite-coated silicon [73]. A decrease in C2C12 cell proliferation on 2 µm pillars patterned on PDMS, PLLA, and a copolymer of PEOT/PBT was seen relative to the respective planar surfaces after 4 days of culture [69]. These studies support the hypothesis that surface topography and chemical composition influence cell proliferation. Furthermore, the spatial arrangement of surface patterns with respect to cell size also appears to be important [59,67]. It has been shown that the amount of space between tantalum pillars with diameters of 1, 2, or 6 µm has a large effect on the cell proliferation of dental-pulp-derived stem cells [67]. Although the interactions between cells and patterned materials is extremely complex, designing the material surface with favorable topological and chemical features can control some aspects of cell behavior.

## Conclusion

In this study, we prepared gelatin with different patterned surfaces using molding and crosslinking with genipin. We then assessed the effect of different surface patterns including grooves, pillars, and holes, with widths or diameters of 2 µm, 1 um, or 500 nm, on Saos-2 cell attachment and proliferation. The gelatin patterned surfaces have a high wettability due to the high gelatin content. It is known that Saos-2 cells prefer to attach to rough surfaces [59,74]. Therefore, Saos-2 cell attachment indicates high roughness on our gelatin patterns with adequate wettability. Our study examining Saos-2 cell proliferation on the different gelatin surface patterns revealed that Saos-2 cells prefer grooves of approximately 2 and 1 µm in width and 500 nm in height, and pillars of 1 µm in diameter and 500 nm in height amongst the different gelatin patterns examined. The number of cells in the pillar group with a 2 µm height was the highest compared to all other groups. The abundant filopodia arising from the Saos-2 cells that were seen to be grasping these pillars might be related to the high degree of cell proliferation (Figure 8e and Figure 9). Although the reason is unclear, gelatin-surface-containing pillars with diameters of 500 nm to 2 µm and height of 2 µm may be of an adequate shape and size for Saos-2 cell proliferation. These data support the concept that a detailed design of surface gelatin patterns can control both Saos-2 cell attachment and proliferation. Furthermore, the softness of the gelatin surface patterns can be controlled by the degree of crosslinking with genipin. Thus, cell behavior can be controlled by changes in the softness of the gelatin. The gelatin patterns crosslinked with genipin will be one of many available biocompatible materials that can be used for surface patterning. This patterning could be used to create an effective seal between soft tissue and dental materials at the surface of an implant or a tooth to protect against microorganism invasion. However, our study suggests that the degree of genipin crosslinking of gelatin can influence cell behavior, and so this key issue remains to be clarified. In addition, further work is underway to clarify why differences in cell attachment and cell proliferation occur between patterned and planar surfaces. As of now, this appears to be a more complicated problem involving surface chemistry, scaffold softness, and cell dynamics, compared to simple changes in surface patterns. Further comparative data studying cell behavior on several different types of collagen/gelatin patterns are required.

## Experimental

### Preparation of gelatin patterns

Cyclo-olefin (COP) or silicone rubber replica molds were prepared according to methods previously reported [11–12,14]. Quartz and silicon master molds were purchased from Kyodo International, Inc. (Kawasaki, Japan). An area of 5 × 5 mm^2^ was patterned as follows: Grooves/ridges, holes, pillars with width or diameter of 2 µm, 1 µm, or 500 nm and a height of 500 nm. In addition, holes with diameter of 2 µm, 1 µm, or 500 nm and a height of 2 µm were also patterned. A 10 × 10 mm^2^ area silicon mold with a hole of diameter of 100 nm and a depth of 200 nm was fabricated at Hokkaido University, Sousei Hall, though the Nanotechnology Platform Japan program. Replicas of the master molds were prepared by heat-pressing COP (ZF14-188, 188 µm thickness, ZEON Corp., Tokyo, Japan) onto the silicon master mold using a compact heating press (AH-1TC, AS ONE Corp., Osaka, Japan) at 165 °C for 4 min under a pressure of 2 MPa. Upon peeling off the COP film, a COP replica mold was obtained. In addition, the PDMS pre-polymer (KE-106 and CAT-RG, 10:1 mix; Shin-Etsu Chemical, Tokyo, Japan) [75] was cast against the above-mentioned replica COP mold and degassed under a vacuum. The PDMS was then heat-cured at 60 °C for 12 h, and then at 100 °C for 3 days. Upon peeling off the cured polymer, a PDMS replica mold with a 0.5 mm thickness was obtained.

**Figure 11:**
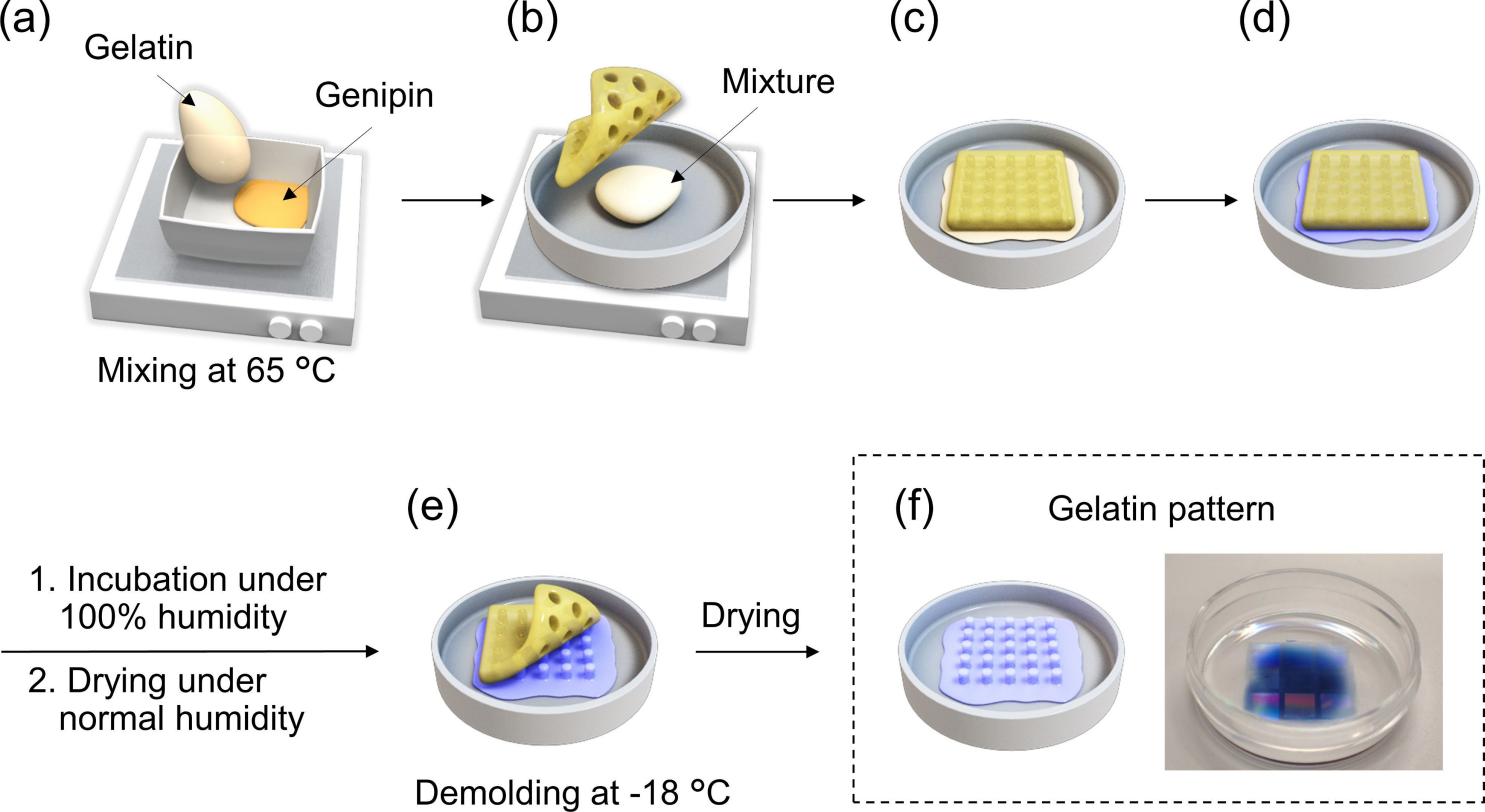
Procedure for the preparation of gelatin patterned surfaces. (a) Addition of a melted gelatin solution to a concentrated genipin solution and mixing at 65 °C, (b) application of the poly(dimethylsiloxane) (PDMS) mold to the mixed solution, (c) gentle pressing of the PDMS mold, (d) incubation for 3 days under 100% humidity at 37 °C, followed by drying for 3 days under normal humidity at 37 °C, (e) demolding at −18 °C and drying, and (f) macroscopic image of the gelatin pattern.

Figure 11 shows the procedure for the fabrication of gelatin-containing micro/nanopatterns using genipin crosslinking. The gelatin powder was obtained from porcine skin (G1890, type A, gel strength: ≈300 g Bloom, isoelectric point: 7–9, Sigma-Aldrich, Tokyo, Japan). First, the gelatin was dissolved at a final concentration of 20 wt % in deionized water at 60 °C. Following this, 9 mg of genipin (Wako Pure Chemical Industries, Inc., Tokyo, Japan) was dissolved in 200 µL ethanol and concentrated at 65 °C. Following this, 2 mL of the 20 wt % gelatin solution was added and mixed for 3 min with the concentrated genipin solution at 65 °C. The final concentration of genipin in the gelatin solution was 20 mM. Next, 225 µL of the mixture was dropped onto a 3.5 cm culture dish and covered with the sterilized PDMS mold. The mold was covered with paper and then manually pressed with a plastic block. The pressed mixture was allowed to cool for 3 min at room temperature after which the paper was peeled off to remove excess gelatin. The remaining gelatin covered with the PDMS mold in the dish was moved to an atmosphere with 100% humidity to avoid drying out and incubated for 3 days at 37 °C. The molds were then dried for 3 days at normal humidity at 37 °C. The molds were then stored at −18 °C to harden the gelatin prior to demolding. Demolding was achieved by carefully peeling off the PDMS mold from the gelatin pattern. The resulting gelatin patterns were then sterilized under UV light for 6 min. Important aspects of the procedure were as follows. The gelatin solution and concentrated genipin solution were mixed for 3 min at 65 °C and immediately applied to the culture dish, and a silicone mold was gently pressed onto the gelatin/genipin mixture. We found that it was important that the viscosity of the solution not be too high, as excessive cross-linking can occur (Figure 11a). In order for continuous crosslinking to occur, the mixture was incubated at 100% humidity in order to avoid drying (Figure 11d-1). The use of a silicone mold allowed water to evaporate from the gelatin/genipin mixture during drying under normal humidity levels (Figure 11d-2). As the gelatin pattern does not fully dry, demolding was carried out at −18 °C to avoid disrupting the gelatin surface pattern (Figure 11e) [76].

### Characterization of the gelatin pattern surface

To understand the effect of genipin concentration on the stability of the gelatin patterns in the culture medium, gelatin solutions were molded and crosslinked using 1, 5, 10, and 20 mM genipin. The resulting gelatin patterns were incubated in DMEM (Sigma-Aldrich, St. Louis, MO, USA) containing 10% FBS (CELLectTM Silver; MP Biomedicals, Solon, OH, USA) at 37 °C in a humidified 5% CO_2_/95% air atmosphere for 1 h, 7 days, or 14 days. For SEM observation, the immersed patterns were fixed with a solution of 2.5% glutaraldehyde for 1 h, and then dehydrated in a graded series of alcohol solutions (50%, 60%, 70%, 80%, 90%, 95%, 99.5%, and 100%) following critical-point drying. Prior to SEM imaging, the patterns were coated with Pt–Pd using a sputtering apparatus (E-1030; Hitachi High-Tech Fielding Corp., Tokyo, Japan). The surface morphology of the patterns was observed using SEM (S-4000; Hitachi High-Tech Fielding Corp., Tokyo, Japan). The surface topography and cross-section of the sputtered pattern was analyzed with a 3D laser scanning confocal microscope (VK-X200; Keyence Corp., Osaka, Japan).

### Evaluation of crosslinking degree

The crosslinking degree of patterned gelatins was determined by using a ninhydrin assay [77]. The ninhydrin solution was prepared according to Yuan’s procedure [78]. The crosslinked gelatins were lyophilized for 2 days. To be dissolved or swelled in water, a 6 mg specimen from different concentrations of genipin was incubated in 200 µm water for 12 h, and then heated at 100 °C for 10 min. Next, 3 mL of the ninhydrin solution was added to the specimen and then heated at 100 °C for 20 min under mild shaking. After cooling, the solution was diluted with 6 mL of 50% isopropanol. The optical absorbance of the further diluted solution was measured with a spectrophotometer (U-1100, Hitachi High-Technologies Corp., Tokyo, Japan) at a wavelength of 570 nm. The crosslinking degree was calculated as the percentage of consumed amino groups in the sample after genipin crosslinking [78]. All data were presented as mean ± standard deviation. Four specimens of each gelatin were used per experiment and the assay was repeated three times to confirm the results.

### Live/dead cell viability assay of Saos-2 cells on gelatin patterns

To estimate the cytotoxicity of gelatin crosslinked with genipin, we carried out a live/dead fluorescence staining assay [79]. Osteoblast-like Saos-2 cells (RCB36888; Riken BioResource Center, Ibaraki, Japan) were seeded at a concentration of 15,000 cells/cm^2^ and cultured for 1 day or 7 days on a gelatin pillar with a diameter of 500 nm and a height of 500 nm at 20 mM genipin on a 3.5 cm dish. The cells were stained with a live/dead double staining kit (Cellstain; Dojindo Laboratories, Kumamoto, Japan). First, the culture medium was removed from the gelatin dish and the gelatin was washed three times with phosphate-buffered saline (PBS). The cells were then stained in 2 mL of the PBS supplemented with 0.7 µM calcein-AM (stained live cells) and 1.4 µM propidium iodide (PI; stained dead cells) in PBS solution for 15 min. The stained cells were observed using a fluorescence microscope (BZ-9000; Keyence Corp., Osaka, Japan). The live and dead cells exhibited green and red fluorescence, respectively. As a control for live cells, cells cultured on TCPS were used (AGC Techno Glass Co., Ltd., Shizuoka, Japan), and as a control for dead cells, cells treated with 70% ethanol for 30 min were used.

### Cell attachment and proliferation assay of Saos-2 cells on different gelatin patterns

To estimate the cell attachment ability of the different gelatin patterns we carried out a cell attachment assay using Saos-2 cells, as previously reported [11,80]. Briefly, gelatin surface patterns in a 3.5 cm tissue culture dish were precoated with 2.5 mL of DMEM containing 10% FBS at 37 °C in a humidified 5% CO_2_/95% air atmosphere for 1 h. After the gelatin surface patterns were washed twice with fresh medium, Saos-2 cells were seeded at a density of 28,000 cells/cm^2^ and incubated on the patterns for 1 h in DMEM containing 10% FBS. After incubation, the patterns were rinsed with PBS to remove non-adhered cells and then fixed with a solution of 2.5% glutaraldehyde and stained with Giemsa dye. Cell attachment was evaluated by optical microscopy and counting of the number of cells attached to each pattern. The values given represent the mean number and standard deviation of the attached cells calculated in a 3.6 mm^2^ area for each pattern. The data are presented as the mean ± standard deviation. Six specimens of each pattern were used per experiment and the assay was repeated twice to confirm the results. For SEM observation of the attached cells, the patterns were fixed with a solution of 2.5% glutaraldehyde, and then dehydrated in a graded series of alcohol solutions (50%, 60%, 70%, 80%, 90%, 95%, 99.5%, and 100%) following critical-point drying. The patterns were then coated with Pt–Pd sputtering and the morphology of the attached cells on the patterns was observed using SEM.

To estimate the cell proliferation ability of Saos-2 cells on the different gelatin surface patterns, we carried out a cell proliferation assay using a similar procedure to that used for cell attachment assay. Briefly, Saos-2 cells were seeded at a concentration of 3,300 cells/cm^2^ and incubated on the patterns for 7 days in DMEM containing 10% FBS. The medium was replaced every 3 days. After incubation, the patterns were rinsed with PBS and then fixed with a solution of 2.5% glutaraldehyde and stained with Giemsa dye. Cell proliferation was evaluated with the procedure described above. The data were presented as mean ± standard deviation of the grown cells calculated in a 3.6 mm^2^ field for each pattern. Six specimens of each pattern were used per experiment and the assay was repeated twice to confirm the results.

### Immunofluorescence staining

The procedure of immunofluorescence staining has been previously described [13]. Briefly, the attached cells on the gelatins were fixed for 5 min in 4% paraformaldehyde in PBS (Wako Pure Chemical Industries, Ltd., Osaka, Japan). The cells were permeabilized with 0.5% Triton X-100 in PBS for 10 min, followed by blocking in 1% bovine serum albumin (BSA) for 30 min. Subsequently, the cells were stained with 500 µL of 1% BSA containing 8 µL of anti-vinculin Alexa-fluor^®^ 488 to detect vinculin (0.5 mg/ml; eBioscience, San Diego, CA, USA); 4 µL of Acti-stain^TM^ 555 fluorescent phalloidin (14 µM; Cytoskeleton Inc., Denver, CO, USA) to detect F-actin; and 3 µL of 4’,6-diamidino-2-phenylindole (DAPI) solution (1 mg/mL; Dojindo Laboratories, Kumamoto, Japan) to detect the nuclei at 37 °C for 1 h, followed by incubation at 4 °C overnight. The specimen was then washed three times in PBS and once in deionized water. Finally, the specimen was mounted with ProLong^®^ Diamond antifade mount reagent (Thermo Fisher Scientific Inc., Tokyo, Japan) and examined with a fluorescence microscope (Biorevo BZ-9000; Keyence Corp., Osaka, Japan).

### Statistical analysis

Statistical analysis was performed using GraphPad Prism version 6.04 (GraphPad Software, Inc., La Jolla, CA, USA). All data are presented as the mean ± standard deviation. Statistical differences were assessed by one-way ANOVA with Tukey’s multiple comparison post-hoc test. A value of *p* < 0.05 was considered statistically significant.
